# Effects of passive, active, and mixed playing strategies on external and internal loads in female tennis players

**DOI:** 10.1371/journal.pone.0239463

**Published:** 2020-09-22

**Authors:** Matthias W. Hoppe, Thilo Hotfiel, Alexandra Stückradt, Casper Grim, Olaf Ueberschär, Jürgen Freiwald, Christian Baumgart

**Affiliations:** 1 Institute of Movement and Training Science I, University of Leipzig, Leipzig, Germany; 2 Center for Musculoskeletal Surgery Osnabrück (OZMC), Klinikum Osnabrück, Osnabrück, Germany; 3 Department of Orthopedic Surgery, Friedrich-Alexander-University Erlangen-Nuremberg, Erlangen, Germany; 4 Department of Movement and Training Science, University of Wuppertal, Wuppertal, Germany; 5 Institute for Applied Training Science (IAT), Leipzig, Germany; 6 Chair for Human-Machine Interaction, Magdeburg-Stendal University of Applied Sciences, Magdeburg, Germany; Eötvös Loránd University, HUNGARY

## Abstract

This study aimed to investigate the effects of different playing strategies on external and internal loads in female tennis players during match play. Also, the underlying effects on the technical-tactical actions and activity profiles were examined. Twelve well-trained female players (age: 25±5 years; maximum oxygen uptake: 40.9±4.3 ml/kg/min) played points against an opponent of similar ability outdoors on red-clay courts. The players played points over five playing conditions. Before each condition, the players were instructed to apply either a passive, an active, or their own playing strategy (free play) to succeed. The five conditions were played in a randomized order, whereas the condition with the own strategy was always played first and served as control. During play, the external and internal loads were investigated by 10 Hz global positioning system, 100 Hz inertial measurement unit, short-range telemetry, capillary blood, and visual analog scale procedures. A 25 Hz video camera was used to examine the technical-tactical actions and activity profiles. Compared to the control condition, the passive, active, and mixed playing strategy conditions induce up to large effects on the external loads (running distances with high acceleration and deceleration), up to moderate effects on the internal loads (energy expenditures spent with high metabolic power, lactate concentration, and rating of effort), and up to very large effects on the technical-tactical actions (number of ground strokes and errors) and activity profiles (strokes per rally, rally duration, work to rest ratio, and effective playing time). Our study shows that passive, active, and mixed playing strategies have an impact on the external and internal loads, technical-tactical actions, and activity profiles of female tennis players during match play. This finding should be considered for practical purposes like match analyses and training procedures in the tennis environment.

## Introduction

Tennis match play involves short repeated high-intensity activities over an unpredictable time. The rules of the International Tennis Federation (ITF) mandate that high-intensity periods are separated by recovery intervals of predefined durations [1]. Over the past 20–30 years, tennis has evolved into a physically demanding sport in all age groups and both genders [2]. Keeping pace with this progress requires specific training drills for which knowledge of match play data is essential [3]. In tennis, match play data have been separated into data describing the activity profiles, technical-tactical actions, mechanical power outputs, and physiological responses of the players [4, 5]. Mainly in males, many studies summarized in reviews have investigated the activity profiles and physiological responses [1, 2], whereas only few studies have examined the mechanical power outputs and technical-tactical behaviors [6–12]. This lack of research is surprising, because both aspects strongly determine the multifactorial tennis performance [13, 14].

From a motor control perspective, superior to the technical-tactical behaviors of tennis players are their playing strategies. To explain, technical-tactical behaviors are variable during play and do take the behavior of opponents into account. In contrast, playing strategies are often predefined, self-referential, and thus fixed over a certain or the entire playing time [15]. Although playing strategies are barely defined and investigated in tennis [8, 16], two contrary strategies can be observed in practice: The number of winners is only at top playing levels comparable to that of errors. Thus, a common strategy to succeed is to reduce the own errors by a passive play from the baseline. The contrary strategy is to dominate the rallies by an active play involving powerful topspin strokes at sharp angles across the full court. The goal of this strategy is to force the opponent to errors or to directly win the points by oneself [4, 17].

In 2003, a framework to describe the physical demands of players was introduced. According to external-mechanical or internal-physiological measurable data, it was suggested to differentiate between external and internal loads, respectively [18]. In tennis, external loads have been mainly assessed by positioning systems and inertial measurement units. These technologies allow to quantify distances covered and various speed, acceleration, and deceleration measures [7, 19, 20]. For internal loads, short-range telemetry, portable respiratory gas analyzers, capillary blood samples, and subjective scales have often been used. By these procedures, heart rates, oxygen uptakes, energy expenditures, blood lactate concentrations, and ratings of perceived efforts can be determined [1, 21]. Importantly, external and internal loads are connected by the concept of mechanical efficiency. This means that physical training has either the goal to improve the mechanical power output at a given physiological response or to decrease the physiological response at a given mechanical power output [22]. Consequently, in tennis, only knowledge of both external and internal loads will lead to ideal designed training drills [23].

In tennis, one previous study examined the effects of passive and active playing strategies on only internal loads in male players [16]. The study shows that a passive strategy leads to a higher oxygen uptake, ventilation, heart rate, and blood lactate concentration (all p<0.001) than an active strategy [16]. Recently, we investigated the effects of the two contrary strategies on both external and internal loads compared to a control condition (free play) in female players. The findings show that the passive strategy leads to more distances covered at high acceleration and deceleration, and also to a higher heart rate, blood lactate concentration, and rating of perceived effort (1.1- to 7.2-fold of the smallest worthwhile change). Additionally, the active strategy leads to a lower blood lactate concentration (-2.4-fold), but once more to a higher rating of perceived effort (2.4-fold) [4]. Overall, the previous studies show that passive and active playing strategies have an impact on external and internal loads in tennis.

Unfortunately, both previous studies [4, 16] failed to examine the effect of mixed playing strategy conditions in which passive competed against active players. Further, the vertical work performed during split steps, jumps, or services was not considered to quantify external loads [7]. Additionally, capillary blood lactate and portable respiratory gas procedures were used to investigate metabolic loads. However, during intermittent sports like tennis, blood lactate measures do not reflect metabolic situations at muscular levels [24], are invasive [25], less reliable [26], and allow no real-time monitoring [21]. Concerning the use of portable gas analyzers, it is self-explanatory that they interfere with maximal performances [27]. Last, due to the direct impact of playing strategies on technical-tactical actions [15], it is promising to also investigate the latter during the rallies, which has not been conducted so far. Thus, a study to address all these points is required; especially, in the hitherto barely studied females.

This study aimed to investigate the effects of passive, active, and mixed playing strategies on external and internal loads in female tennis players during match play. Also, the underlying effects on the technical-tactical actions and activity profiles were examined. Based on previous studies [4, 16], it was hypothesized that each playing strategy has a different impact on the external and internal loads, technical-tactical actions, and activity profiles of the players during play. Here, we re-analyze few data of an already published study [4] and evaluate them together with comprehensive unpublished data. Compared to the published study, we (i) analyze not only passive and active, but also mixed playing strategy conditions, (ii) use inertial measurement units to determine external loads more complete, (iii) apply a new metabolic approach, and (iv) quantify ball placements during the rallies as an indicator of the technical-tactical behavior. Our outcomes may increase the understanding of playing strategies in female tennis players, which may be helpful for practical purposes like match analyses and training procedures.

## Materials and methods

### Participants and ethics statement

To test our hypothesis, 12 well-trained female tennis players from local clubs were recruited. The inclusion and exclusion criteria were (i) an age of 20–30 years, (ii) a regional ranking ≤10, (iii) a right-handed stroking technique, and (iv) a balanced combination of baseline play and attacking toward the net. The players were excluded, if there was (v) an acute disease speaking against maximal load testing or (vi) no signed consent to participate. The players were informed of the purposes, procedures, and potential risks of the study. All procedures were pre-approved by the Ethics Committee of the University of Wuppertal (MS/JE 29.11.11) and were conducted in accordance with the Declaration of Helsinki. Table 1 summarizes the anthropometric characteristics and tennis backgrounds of the players.

**Table 1:** Anthropometric characteristics and tennis backgrounds of the female players (n = 12).

Variable	Mean (90% CI)
**Anthropometric characteristics**	
**Age (y)**	25 (22–27)
**Body height (cm)**	167 (164–170)
**Body mass (kg)**	61.0 (58.9–63.1)
**Body fat (%)**	22.4 (20.9–23.9)
**Tennis backgrounds**	
**Regional ranking (1–23)**	5.8 (4.2–7.3)
**Tournaments per season (n)**	18.8 (14.3–23.3)
**Tennis training per week (n)**	2.6 (1.9–3.3)
**Physical training per week (n)**	1.8 (1.6–2.1)

CI = Confidence interval.

### Experimental design

All procedures were conducted on two sessions within one week during the last month of the outdoor season. The players were asked to report to both sessions well rested, to refrain from strenuous exercise for the prior 24 hours, and to prepare themselves as they would for an official competition. On the first session, the players were examined on a motorized treadmill for maximal oxygen uptake and heart rate in the laboratory. On the second session, the players were tested on court. The data collection took place outdoors on a red-clay court at 22–26°C and 38–45% humidity. After the players had warmed-up for 10 min with ground strokes, volleys, overhead strokes, and serves, they were asked to play points against an opponent of similar ability. The opponents were matched by a professional tennis coach, who was familiar with the players. The service changed after one player had served from both sides. During play, the players retrieved their own balls and counted the won points.

The players played points over five playing conditions per 10 min. The conditions were separated by 5 min rest periods. Before each condition, the players were instructed to apply either: (i) a passive, (ii) an active, or (iii) their own playing strategy to succeed, as previously conducted [4]. The instructions were given on cards and formulated in an open manner. They read: (i) “try to win the points through a reduction of your own errors”, (ii) “try to win the points directly by yourself or by forcing the opponent to errors”, and (iii) “try to win the points with your own strategy that you would also apply in a competition”. The players were instructed not to communicate about the content of their received instructions. While the players applied the same strategies during three conditions (labeled as “both own”, “both passive”, and “both active”), they applied mixed strategies during two conditions (labeled as “mixed passive” and “mixed active”). For the mixed conditions, the data were analyzed from the perspectives of the passive and active players, respectively. Each pair of players played the five conditions in a randomized order, whereas the condition with the own strategy was always played first and served as control. After 5 min of play, the players were verbally reminded between two rallies by the tennis coach to consider their attained instructions. The last rally was played out.

**Fig 1:**
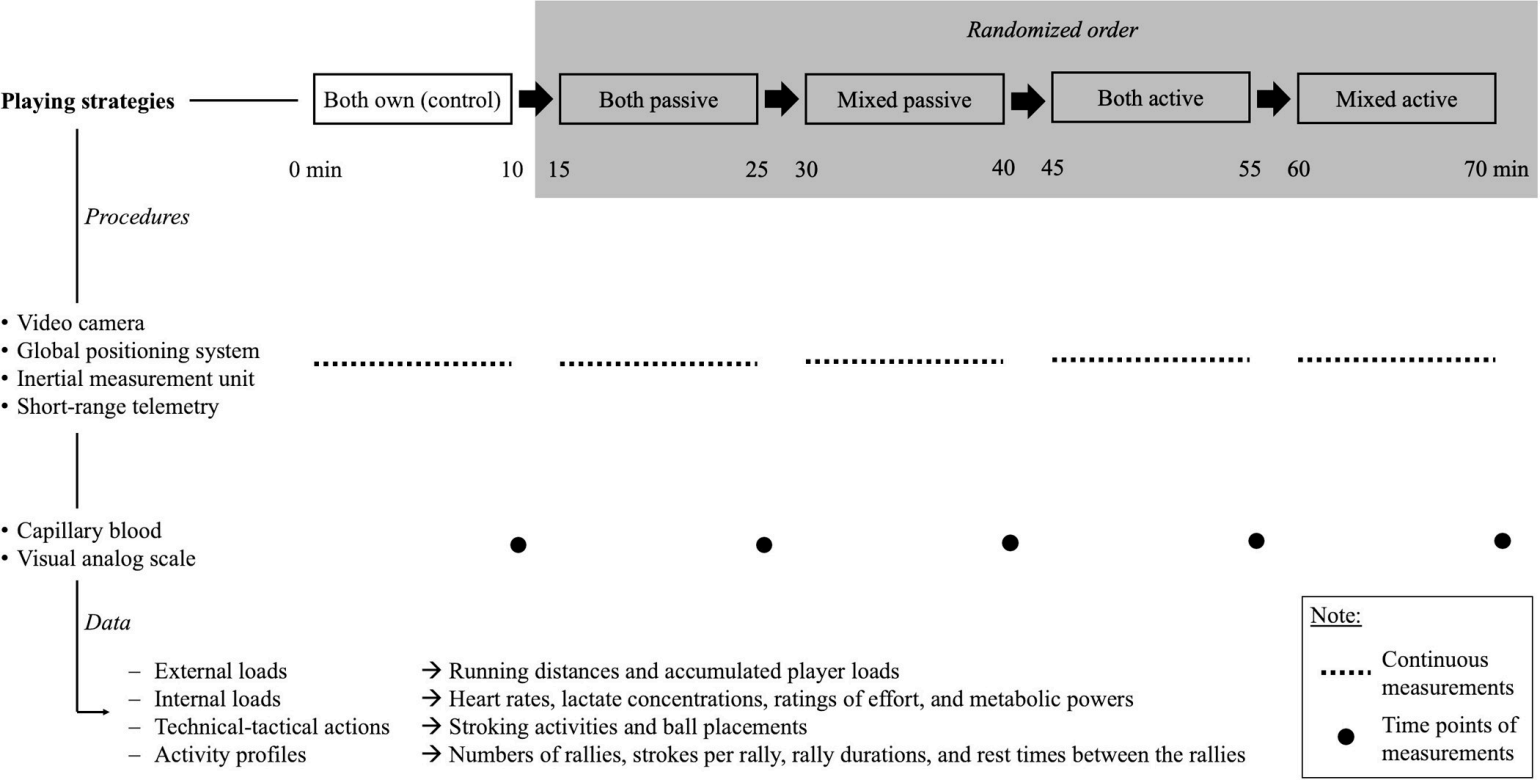
Design of the on-court testing session.

To estimate the external and internal loads, and also the underlying technical-tactical actions and activity profiles, global positioning system, inertial measurement unit, short-range telemetry, capillary blood, visual analog scale, and video camera procedures were applied. While the capillary blood and visual analog scale procedures were applied at the beginning of the rest periods, all other procedures were continuously applied during the five playing conditions. Fig 1 displays the design of the on-court testing session.

An overview of the data that were operationalized to reflect the external and internal loads, technical-tactical actions, and activity profile of the female tennis players during match play is also given.

### Laboratory testing

To determine the maximal oxygen uptake and heart rate, an incremental running test on a motorized treadmill (H/P Cosmos, Pulsar, Nussdorf-Traunstein, Germany) was performed, as previously described [28]. Briefly, during the test, respiratory gas exchanges and heart rates were measured using an open-circuit breath-by-breath gas analyzer (Ganshorn, PowerCube-Ergo, Niederlauer, Germany) and short-range telemetry (Polar, T31, Kempele, Finland), respectively. The collected data were averaged over 10 s. Before each test, the gas analyzer was calibrated with a calibration gas (15.5% O_2_, 5% CO_2_ in N; Messner, Switzerland) and a precision 1-liter syringe (Ganshorn, Germany) according to the instructions of the manufacturer. The initial two increments of the protocol consisted of running for 4 min at 10 km/h at an inclination of 1% and 5%, respectively. Thereafter, the speed was increased every 2 min by 1 km/h until exhaustion was reached. The exhaustion was considered to be reached, if a plateau in oxygen uptake (increase <2 ml/kg/min) despite an increase in the workload was observed. Otherwise, three of the following four criteria had to be fulfilled: (i) a heart rate ≥95% of an age-predicted maximal heart rate (220-age), (ii) a respiratory exchange ratio ≥1.15, (iii) a capillary blood lactate concentration ≥8.0 mmol/l, or (iv) a Borg rating of perceived exertion ≥19. The maximal oxygen uptake and heart rate were defined as the highest recorded data during the workload. Before testing, anthropometric and body composition data were assessed. The body fat was predicted by a 4-point bioelectric impedance analysis (Bodystat, QuadScan 4000, Douglas, United Kindgom) in supine position.

### On-court testing

#### External loads

To estimate the external loads, portable combined 10 Hz global positioning systems and 100 Hz inertial measurement units (MinimaxX S4, Catapult Innovations, Melbourne, Australia) were used. The global positioning system measured running speed data by the Doppler-shift, whereas the inertial measurement unit assessed acceleration data in all three movement planes by triaxial accelerometers [29]. The devices were worn beneath the players’ attire in custom-made neoprene harnesses located between the scapulae. To allow for the satellite lock, the devices were activated 15 min prior to the data collection. The collection took place under a cloudless sky between 09.00–11.00 A.M. and there were no tall buildings or trees around the court that could have had negative influences on signal qualities. During play, the devices had connections with ≥9 satellites and the horizontal dilution of position was ≤0.92, indicating ideal measurement conditions [30]. The reported raw data were exported from the proprietary software (Sprint, version 5.1.4, Catapult Innovations, Melbourne, Australia) to custom-made spreadsheets that incorporated macro-based calculations (Microsoft, Excel 2016, Redmond, WA, USA) for further analyses.

To minimize noise, the running speed data assessed by the global positioning systems were proceeded by a second order low-pass Butterworth filter using a cut-off frequency of 1 Hz and two passes. From the filtered speed data and their integration over the time, the total running distances and those with low (<3 m/s) and high speed (≥3 m/s) were computed. Additionally, the filtered speed data were deviated over the time to derive acceleration and deceleration data. To also minimize noise from these data, they were filtered by a slightly modified filter (instead of 1 Hz with 0.5 Hz). Then, from the filtered data, the running distances with low (<2 m/s²) and high acceleration (≥2 m/s²) as well as low (>-2 m/s²) and high deceleration (≤-2 m/s²) were computed. Finally, from the acceleration data measured by the inertial measurement units in all three movement planes, the accumulated player loads were computed. The player load is one of the most common accelerometer derived parameter used to quantify total acceleration based external loads [31] and was calculated according to the following equation (Eq 1) [29]:

$${\text{Player}\;\text{load}}_{\text{t}=\text{n}}=\;\sum_{\text{t}=0}^{\text{t}=\text{n}}\sqrt{\left(\left({\text{fwd}}_{\text{t}=\text{i}+1}-{\text{fwd}}_{\text{t}=\text{i}}\right)²+\left({\text{side}}_{\text{t}=\text{i}+1}-{\text{side}}_{\text{t}=\text{i}}\right)²+\left({\text{up}}_{\text{t}=\text{i}+1}-{\text{up}}_{\text{t}=\text{i}}\right)²\right)}$$ (1),

whereas fwd, side, and up is forward, sideway, and upward acceleration, respectively. All filtering techniques, threshold definitions, and computational steps were applied according to previous tennis research [6].

#### Internal loads

To estimate the internal loads, heart rate, capillary blood lactate concentration, rating of effort, and metabolic power data were examined. The heart rate data were assessed at 1 Hz by short-range telemetry (Polar, T31, Kempele, Finland). The collected data were analyzed in relation to the maximal values achieved during the laboratory treadmill tests. They were considered as low (<85%) and high heart rates (≥85%) according to previous tennis research [32]. The lactate concentrations were determined from 20 μl capillary blood samples by an electro-enzymatic analyzer (EKF-diagnostics, Biosen C_line Sport, London, United Kingdom). From each blood sample, the concentrations were determined in duplicate and the mean was recorded. The ratings of efforts were quantified by 100 mm visual analogue scales.

Beside these standard procedures to estimate internal loads in tennis, metabolic power data were also investigated. The metabolic power is defined as the instantaneous muscular energy demand that is required to maintain the ATP level constant [33]. To compute, the filtered running speed, acceleration, and deceleration data collected by the global positioning systems were used for an equivalent slope approach, as described in detail elsewhere [34]. Briefly, accelerated and decelerated running on a horizontal level is energetically equivalent to uphill and downhill running at a constant speed on an equivalent slope, whereby the slope is dictated by the forward acceleration and deceleration. Since the energetics of uphill and downhill running are well known and energy costs are independent of the speed and cluster on average about 4.0 J/kg/m, the energy costs of accelerated and decelerated running on a horizontal level can be estimated. The energy cost can then be multiplied by the underlying speed to calculate the instantaneous metabolic power [34]. For all computational steps, the original equations were used [35] that can be simplified as follows (Eq 2):

$$Metabolic\;power\;\left(t\right)=\left(\left(155.4ES{\left(t\right)}^{5}-30.4ES{\left(t\right)}^{4}-43.3ES{\left(t\right)}^{3}-46.3ES{\left(t\right)}^{2}+19.5ES\left(t\right)+4.0\right)\times {\left({\left(\frac{a\left(t\right)}{g}\right)}^{2}+1\right)}^{0.5}\times KT\right)\times v\left(t\right)$$ (2),

whereas ES is equivalent slope, 4.0 is energy cost for running at a constant speed in J/kg/m, a is forward acceleration, g is acceleration due to gravity, KT is a terrain constant of 1.29, and v is speed. The equivalent slope was computed accordingly (Eq 3) [34]:

$$Equivalent\;slope\;\left(t\right)=tan\left(90-arctan\left(\frac{g}{a\left(t\right)}\right)\right)$$ (3),

whereas tan and arctan are tangent and arctangent, respectively.

The computed metabolic power data were applied to the subsequent analyses: First, from the time integral of the metabolic power data, the energy expenditures were computed. Then, the energy expenditures spent with low and high metabolic power were calculated [36]. In this context, we [7] and a further research group [37] previously analyzed metabolic power data during tennis match play based on absolute thresholds. This can be seen as a limitation, because it is clear that metabolic capacities differ between the players. Thus, there is a need to develop individualized metabolic power thresholds [33]. Since metabolic power data can be converted into oxygen uptake units, one rational possibility for an individualization is to analyze the metabolic power data with respect to the maximal power of the aerobic system–that is the maximal oxygen uptake [36]. To implement this approach for the first time, the maximal oxygen uptakes achieved during the laboratory treadmill tests were converted into corresponding individual metabolic power thresholds as follows (Eq 4):

$$Individual\;metabolic\;power\;threshold=\;\frac{Maximal\;oxygen\;uptake}{2.87}$$ (4),

whereas individual metabolic power threshold is in W/kg and maximal oxygen uptake is in ml/kg/min. The conversion assumes a respiratory exchange ratio of 0.96 for intermittent sports like tennis and a corresponding energy equivalent of 20.9 kJ per liter of oxygen uptake [36]. Plausibly, the outcomes of our individualization allow to differ between energy supplies from predominantly aerobic (metabolic power <maximal oxygen uptake; labeled as “low metabolic power”) and anaerobic sources (metabolic power ≥maximal oxygen uptake; labeled as “high metabolic power”) [36], which has not been conducted so far.

#### Technical-tactical actions and activity profile

**Fig 2:**
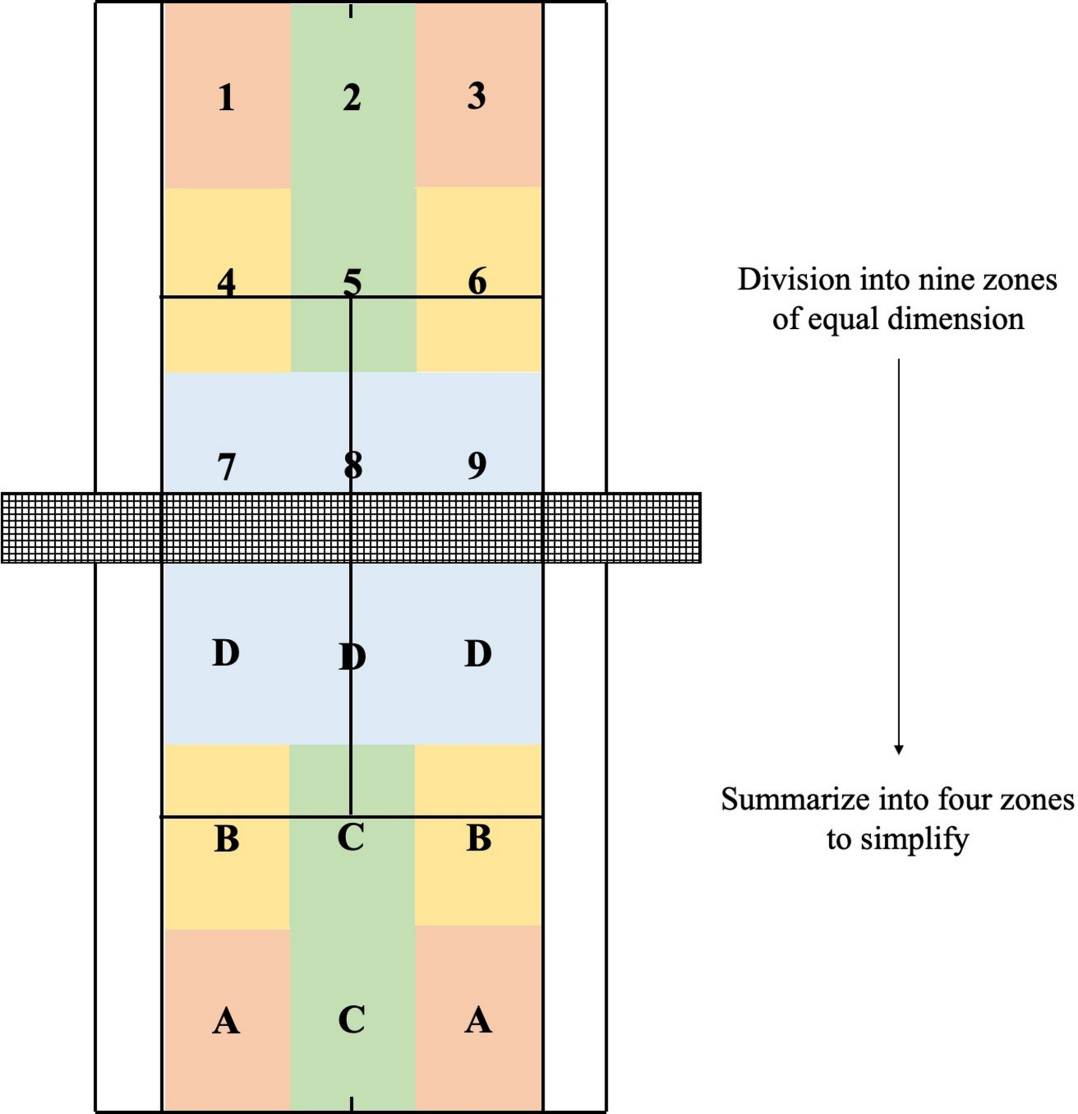
Analyzed ball placements of the ground strokes in the four different zones (labeled from “A” to “D”) on the tennis court. First, the tennis court was divided into nine zones of equal duration (upper part). Then, the nine zones were summarized into four zones to simplify (lower part). See text for further details.

To also estimate the underlying technical-tactical actions and activity profiles, video data were assessed by a 25 Hz camera (DCR-SR190, Sony, Tokyo, Japan). The camera was positioned 10 m behind the baseline of the court at 10 m above the ground. Using an open-source software (Kinovea, version 0.8.15.), the following technical-tactical actions were determined from the video data according to previous tennis research [4]: relative rate of 1^st^ services, numbers of double faults, number of ground strokes as well as relative rates of forehands, backhands, volleys, winners, and errors. Additionally, the ball placements of the ground strokes were analyzed. Therefore, the tennis court was divided into nine zones of equal dimension. Then, the nine zones were summarized into four zones with the following meanings to simplify: (i) “into the corners of the court” (labeled as “zone A”), (i) into the middle of the court and next to the side lines” (labeled as “zone B”), (iii) “into the center of the court” (labeled as “zone C”), and (iv) “just behind the net” (labeled as “zone D”). The four zones were chosen, because it was expected that the different playing strategy conditions would lead to different ball placements therein. Fig 2 shows the analyzed ball placements of the ground strokes in the four different zones on the tennis court.

Finally, the video data were also used to determine the activity profiles. According to previous tennis research [38], the following data were assessed: number of rallies, strokes per rally, rally duration, rest time between the rallies, work to rest ratio, and effective playing time. A professional tennis coach performed all video analyses. From one randomly selected player and a second skilled observer, the intra- and inter-rater reliabilities of all our video analyses were determined. Our reliability outcomes (CV<2.2%) were comparable to those of previous tennis research (CV<1.9%) [39].

### Statistical analysis

For the statistical analysis, Magnitude-Based Inferences were computed, as described in detail elsewhere [40]. Briefly, means and 90% confidence intervals were computed first. Then, the dispositions of the confidence intervals for the effects in relation to the smallest worthwhile changes were examined. The smallest worthwhile changes were computed from the pooled standard deviations multiplied by 0.2. Finally, the probabilities of the effects for being higher, similar, or lower than the smallest worthwhile changes were determined and qualitatively described using the following probabilistic scale: <0.5%, most unlikely; 0.5 to <5%, very unlikely; 5 to <25%, unlikely; 25 to <75%, possibly; 75 to <95%, likely; 95 to <99.5%, very likely; and ≥99.5%, most likely. If the probabilities of the effects for being both higher and lower than the smallest worthwhile changes were ≥5%, the effects were described as unclear. To clarify the magnitudes of the effects, standardized differences labeled as effect sizes were calculated and interpreted accordingly: 0.2 to <0.6, small; 0.6 to <1.2, moderate; 1.2 to <2.0, large; 2.0 to <4.0, very large; and ≥4.0, extreme large. For the visualization in panels, all effects were shown as factors of the smallest worthwhile changes with their corresponding effect sizes.

## Results

Table 2 summarizes the exhaustion criteria and main outcomes of the players during the incremental running tests. On average, the defined exhaustion criteria were fulfilled. Thus, the main outcomes regarding maximal data can be considered as valid. The mean maximal oxygen uptakes and heart rates were 40.9 ml/kg/min and 190 bpm, respectively. The corresponding mean metabolic power and heart rate thresholds were 14.3 W/kg and 162 bpm, respectively.

**Table 2:** Exhaustion criteria and main outcomes of the female tennis players (n = 12) during the incremental running tests.

Variable	Mean (90% CI)
**Exhaustion criteria**	
**Plateau in oxygen uptake (n)**	5 of 12 players
**Respiratory exchange ratio (VCO**_**2**_**/VO**_**2**_**)**	1.22 (1.19–1.25)
**Lactate concentration (mmol/l)**	10.3 (9.4–11.2)
**Rating of perceived exertion (6–20)**	18.8 (18.4–19.2)
**Main outcomes**	
**Maximal oxygen uptake (ml/kg/min)**	40.9 (38.9–43.0)
Metabolic power threshold*(W/kg)	14.3 (13.5–15.0)
Maximal heart rate** (bpm)	190 (187–194)
Heart rate threshold* (bpm)	162 (159–165)

CI = Confidence interval; VCO_2_ = Carbon dioxide; VO_2_ = Oxygen uptake;

* = Both thresholds were computed to distinguish between low and high metabolic power and heart rate during tennis match play;

** = The maximal heart rate was also used as an exhaustion criterion. See text for further details.

Table 3 summarizes all descriptive data for the external and internal loads, technical-tactical actions, and activity profiles during match play according to the passive, active, and mixed playing strategy conditions.

**Table 3:** Descriptive data for the external and internal loads, technical-tactical actions, and activity profile of the female tennis players (n = 12) during match play according to the passive, active, and mixed playing strategy conditions.

Variable	Mean (90% CI)				
	Both own (control)	Both passive	Mixed passive	Both active	Mixed active
**External loads**					
**Total distance (m)**	396 (363–429)	423 (390–455)	421 (399–442)	397 (366–429)	401 (382–419)
**Low speed (m)**	392 (361–423)	418 (388–449)	410 (394–426)	392 (362–423)	394 (376–413)
**High speed (m)**	4 (1–6)	5 (2–7)	11 (8–14)	5 (4–6)	6 (4–8)
**Low acceleration (m)**	244 (225–263)	262 (240–284)	256 (245–267)	245 (226–265)	246 (232–259)
**High acceleration (m)**	11 (9–13)	18 (16–20)	14 (12–17)	12 (9–14)	12 (11–14)
**Low deceleration (m)**	115 (102–128)	116 (105–126)	120 (113–126)	116 (105–127)	116 (109–122)
**High deceleration (m)**	5 (4–7)	6 (5–7)	11 (9–12)	5 (4–6)	5 (5–6)
**Player load (a.u.)**	52.5 (52.3–52.7)	64.7 (64.3–65.1)	52.4 (52.2–52.7)	49.6 (49.4–49.9)	53.8 (53.6–54.0)
**Internal loads**					
**Low heart rate (s)**	400 (317–483)	328 (248–408)	416 (346–486)	447 (376–518)	398 (324–471)
**High heart rate (s)**	210 (130–291)	292 (211–372)	196 (125–267)	169 (99–239)	214 (141–286)
**Lactate concentration (mmol/l)**	1.8 (1.5–2.2)	2.3 (2.0–2.7)	1.6 (1.3–2.0)	1.5 (1.3–1.7)	1.8 (1.4–2.1)
**Energy expenditure (J/kg)**	1746 (1569–1923)	1941 (1834–2047)	1854 (1709–2000)	1748 (1578–1918)	1792 (1719–1866)
**Low metabolic power (J/kg)**	1537 (1410–1663)	1611 (1531–1691)	1575 (1489–1661)	1554 (1428–1680)	1570 (1511–1629)
**High metabolic power (J/kg)**	209 (149–269)	330 (282–379)	279 (210–348)	194 (138–250)	223 (184–262)
**Rating of effort (0–100)**	23 (14–31)	36 (22–49)	35 (24–46)	34 (23–44)	38 (27–50)
**Technical-tactical actions**					
**1**^**st**^ **services (%)**	69.8 (68.4–71.1)	72.0 (69.6–74.5)	77.0 (72.7–81.4)	68.7 (66.5–70.9)	71.6 (66.3–76.9)
**Double faults (n)**	2.3 (1.7–3.0)	1.3 (0.7–2.0)	0.6 (0.3–0.9)	3.2 (2.5–3.8)	0.8 (0.5–1.0)
**Ground strokes (n)**	53.7 (48.8–58.5)	121.0 (105.1–136.9)	31.8 (27.3–36.3)	45.2 (37.0–53.4)	32.4 (27.5–37.3)
**Forehands (%)**	53.3 (47.2–59.5)	53.3 (47.9–58.7)	51.1 (45.9–56.4)	51.5 (47.8–55.2)	54.3 (47.4–61.3)
**Backhands (%)**	46.1 (39.7–52.4)	46.2 (40.9–51.6)	48.3 (42.9–53.7)	45.4 (42.0–48.8)	42.3 (35.4–49.2)
**Volleys (%)**	0.6 (0.2–1.1)	0.4 (0.1–0.8)	0.6 (-0.1–1.2)	3.1 (1.7–4.6)	3.4 (1.6–5.1)
**Winners (%)**	6.3 (4.8–7.9)	2.1 (1.5–2.7)	3.3 (1.8–4.7)	10.3 (9.2–11.5)	12.6 (8.9–16.4)
**Errors (%)**	28.4 (26.1–30.7)	12.7 (10.4–15.0)	18.5 (13.1–23.9)	40.6 (32.0–49.1)	34.6 (26.2–43.0)
**Ball placements zone A (%)**	35.8 (33.5–38.2)	36.6 (34.2–38.9)	25.3 (18.5–32.1)	37.6 (34.4–40.8)	40.3 (37.4–43.1)
**Ball placements zone B (%)**	30.5 (30.1–31.0)	29.4 (25.8–33.1)	23.6 (18.3–28.9)	31.2 (29.9–32.5)	33.4 (29.
**Ball placements zone C (%)**	30.0 (27.9–32.1)	31.2 (27.3–35.0)	46.2 (40.2–52.1)	29.8 (26.3–33.2)	23.0 (19.1–26.9)
**Ball placements zone D (%)**	3.7 (2.6–4.8)	2.8 (1.9–3.7)	4.9 (2.7–7.2)	1.4 (0.8–2.0)	3.3 (2.0–4.6)
**Activity profile**					
**Number of rallies (n)**	19.2 (18.4–19.9)	16.0 (15.2–16.8)	20.7 (19.2-22-1)	20.2 (19.3–21.0)	21.0 (19.4–22.6)
**Strokes per rally (n)**	4.7 (4.4–4.9)	9.7 (8.4–11.0)	5.0 (4.5–5.5)	4.1 (3.7–4.4)	5.0 (4.3–5.7)
**Rally duration (s)**	6.8 (6.4–7.2)	16.9 (14.3–19.5)	7.7 (6.6–8.8)	5.5 (4.9–6.2)	7.8 (6.5–9.2)
**Rest time between rallies (s)**	26.1 (24.6–27.5)	22.9 (21.2–24.7)	23.2 (21.4–24.9)	25.6 (24.1–27.1)	22.6 (20.7–24.6)
**Work to rest ratio**	3.9 (3.5–4.3)	1.6 (1.2–2.0)	3.2 (2.8–3.6)	5.0 (4.2–5.8)	3.2 (2.7–3.7)
**Effective playing time (%)**	21.4 (19.8–23.0)	42.8 (37.2–48.4)	25.4 (23.0–27.8)	18.2 (15.8–20.6)	26.0 (22.5–29.6)

CI = Confidence interval.

**Fig 3:**
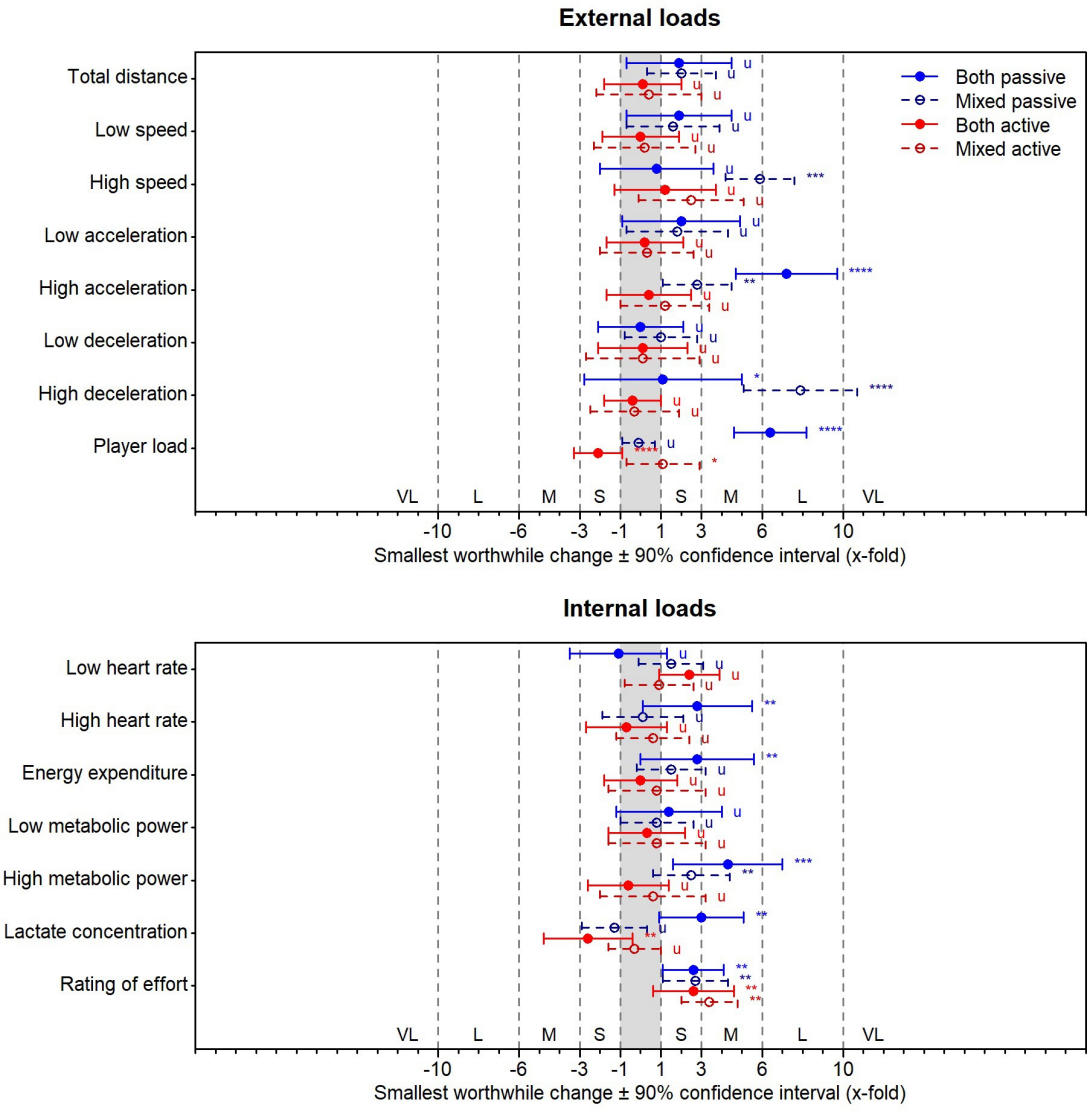
Effects of the passive, active, and mixed playing strategy conditions on the external (upper panel) and internal loads (lower panel) in the female tennis players (n = 12) during match play. The effects are shown as factors of the smallest worthwhile changes. The corresponding effect size thresholds for small (S; ±1-fold), moderate (M; ±3-fold), large (L; ±6-fold), and very large effects (VL; ±10-fold) are also shown. The asterisks *, **, ***, and **** indicate the probabilities that the effects are possibly (≥25%), likely (≥75%), very likely (≥95%), and most likely (≥99.5%) higher or lower than the smallest worthwhile changes. The letter u indicates unclear effects with probabilities of ≥5% that the effects are both higher and lower than the smallest worthwhile changes. See text for further details.

**Fig 4:**
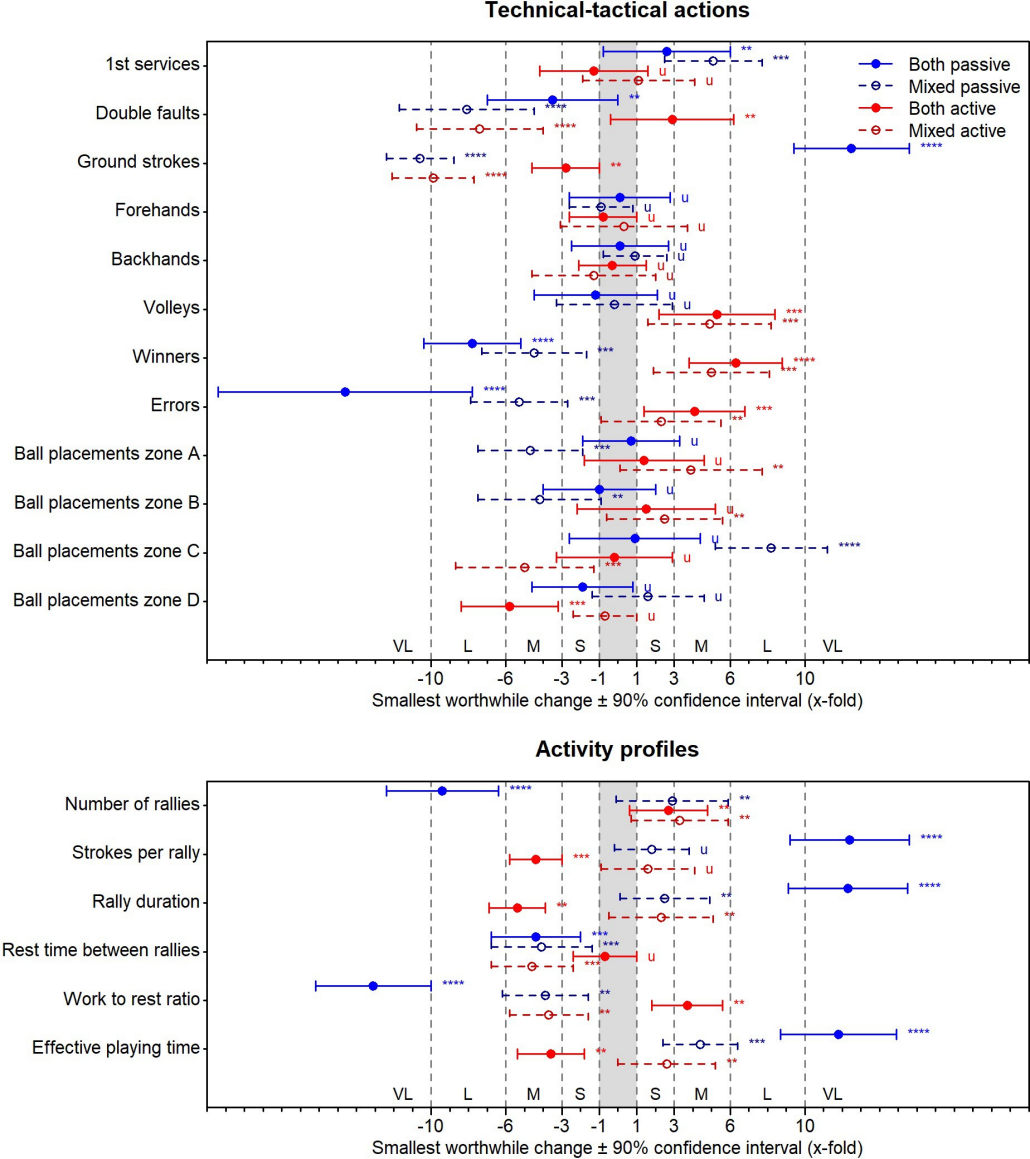
Effects of the passive, active, and mixed playing strategy conditions on the technical-tactical actions (upper panel) and activity profile (lower panel) in the female tennis players (n = 12) during match play. The effects are shown as factors of the smallest worthwhile changes. The corresponding effect size thresholds for small (S; ±1-fold), moderate (M; ±3-fold), large (L; ±6-fold), and very large effects (VL; ±10-fold) are also shown. The asterisks *, **, ***, and **** indicate the probabilities that the effects are possibly (≥25%), likely (≥75%), very likely (≥95%), and most likely (≥99.5%) higher or lower than the smallest worthwhile changes. The letter u indicates unclear effects with probabilities of ≥5% that the effects are both higher and lower than the smallest worthwhile changes. See text for further details.

Fig 3 shows the effects of the passive, active, and mixed playing strategy conditions on the external and internal loads during match play. Fig 4 displays the effects on the technical-tactical actions and activity profiles.

Compared to the control condition, the both passive playing strategy condition exhibits up to large effects on the external loads (increased running distances with high acceleration and player load), up to moderate effects on the internal loads (increased energy expenditures spent with high metabolic power and lactate concentration), up to very large effects on the technical-tactical actions (increased number of ground strokes and decreased number of errors), and up to very large effects on the activity profiles (increased strokes per rally, rally duration, and effective playing time and decreased work to rest ratio).

The both active condition induces up to small effects on the external loads (decreased player load), up to small effects on the internal loads (decreased lactate concentration and increased rating of effort), up to large effects on the technical-tactical actions (increased number of winners), and up to moderate effects on the activity profiles (decreased strokes per rally, rally duration, and effective playing time and increased work to rest ration).

The mixed passive condition displays up to large effects on the external loads (increased running distance with high deceleration), up to small effects on the internal loads (increased energy expenditures spent with high metabolic power and rating of effort), up to very large effects on the technical-tactical actions (decreased number of ground strokes), and up to moderate effects on the activity profiles (decreased rest time between the rallies and work to rest ratio and increased effective playing time).

The mixed active condition demonstrates up to small effects on the external loads (increased player load), up to moderate effects on the internal loads (increased rating of effort), up to large effects on the technical-tactical actions (reduced number of double faults and ground strokes), and up to moderate effects on the activity profiles (increased number of rallies and decreased rest time between the rallies and work to rest ratio).

## Discussion

This study aimed to investigate the effects of passive, active, and mixed playing strategies on external and internal loads in female tennis players during match play. Also, the underlying effects on the technical-tactical actions and activity profiles were examined. As hypothesized, our key finding was that each playing strategy has a different impact on the external and internal loads, technical-tactical actions, and activity profiles of the players during play. This outcome is supported by the few available studies that have also investigated the effects of playing strategies [4, 16] or that of further strategical situations as service and return games [38, 41] on tennis match play data. Consequently, playing strategies should be considered for practical purposes like match analyses and training procedures in the tennis environment.

The tennis performance depends on interdependent relationships between anthropometric characteristics, physical capacities, technical-tactical skills, psychological factors, and medical aspects [2, 42]. Due to this complexity, numerous factors have an impact on tennis match play data. The latter can be assessed on four different categories: (i) external loads, (ii) internal loads, (iii) technical-tactical actions, and (iv) activity profiles [4, 5]. In tennis, many studies investigated the internal loads and activity profiles. These studies show that factors like the age, sex, playing level, playing surface, ball type, or thermal condition have an impact on both data levels [1, 2]. Our study is the first to demonstrate that playing strategies have an up to very large impact on all four match play data levels (Figs 3 and 4). Therefore, playing strategies should be considered as a further factor that influences match play data in tennis.

In tennis, match analyses can yield helpful data for the training process [3]. However, an understanding of the interrelations between the different match play data categories is crucial [1, 2]. Our study shows that the largest effects of the playing strategies were evident on technical-tactical actions (Fig 4). This outcome supports the assumed association between both, namely that playing strategies are straight superior to technical-tactical actions from a motor control perspective [15]. Additionally, the sizes of our found effects indicate that changes in the technical-tactical actions may then lead to changes in the activity profiles (Fig 4), and finally in the external and internal loads (Fig 3). Thus, for match analyses concerning external and internal loads in tennis, an integrated approach that takes the underlying strategical and technical-tactical context into account is needed; especially, to transfer the findings into the practice [43]. For example, external and internal load measures during match play can be used to establish profiles of adult tennis players. Then, these profiles can serve as a training framework to develop younger players in terms of the physical needs to play at an adult age. However, our study question such an approach in tennis and support rather the idea that playing strategy specific profiles should be considered; of course, with respect to further crucial context factors like the playing surface [37] or service and return situation [41].

Presently, tennis is a physically demanding sport. To fulfill the playing demands [44], and thereby, to also prevent potential overuse injuries for instance of the shoulder [45], specific training drills are required. Therefore, and supported by a tennis review [2], our results propose that playing strategies should be considered, because each of our investigated strategy has a different impact on the external and internal loads, technical-tactical actions, and activity profiles of the players during play (Figs 3 and 4). In fact, the two passive playing strategy conditions lead to higher external and internal loads (Fig 3). Specifically, the both passive condition leads to higher cardiovascular and metabolic demands, whereas the mixed passive condition leads to higher muscular demands, as indicated by our speed, acceleration, heart rate, metabolic power, and blood lactate concentration measures (Fig 3). On the contrary, the two active conditions lead to more skilled technical-tactical actions, as indicated by the stroking activities and ball placements (Fig 4). Although both active conditions differ only less in external and internal loads and technical-tactical actions, it is noteworthy that they lead to opposed effects on the activity profiles (Figs 3 and 4).

Not only for physical, but also for technical-tactical training purposes, our findings indicate that drills should consider the preferred or intended playing strategies of the players. For example, in players that favor passive strategies, the focus should be placed on intermittent endurance capacities [2] and skills to reduce unforced errors [17]. In contrast, for players with active strategies, the focus should be placed on muscular power capacities [2] and skills to perform topspin strokes at sharp angles across the full court [7]. Because playing strategies can change during play according to the circumstance of matches [15], for example after the first set in which a predefined strategy was not successful, and many players favor a mixture of passive and active strategies today [1], the given global training recommendations should be considered variable and with different foci. For example, within off- and on-court training drills during the pre-season, between the tournaments as preparations for the next playing surface, or during the rehabilitation after injuries and illnesses.

While our study increases the knowledge of playing strategies in the tennis environment, few limitations have to be acknowledged. First, the effects of playing strategies on further outcomes like match results [6] or fatigue statuses [46] of the players remain unknown. Second, our relatively small sample size limits a generalization of the findings and we did also not perform a sample size calculation a priori. However, it should be considered that we aimed to conduct a comprehensive methodological approach to allow a holistic understanding of the examined relationships and used statistical calculations that are independent of the number of observations [47]. Third, a transfer of our outcomes to real match conditions is limited, because simulated matches were investigated here [48]. Thus, further studies that address these points are required. Future studies should also investigate tennis players from various backgrounds, especially according to sex, age, and playing level, as well as external and internal loads in a more specific manner. For instance, concerning different hard and soft tissue loads or molecular responses for which new inertial measurement units [49] and capillary blood [28] based approaches are promising.

## Conclusion

In conclusion, our study is the first to show that passive, active, and mixed playing strategies have a different impact on the external and internal loads, technical-tactical actions, and activity profiles of female tennis players during match play. This finding should be considered for practical purposes like match analyses and training procedures in the tennis environment.

## Supporting information

S1 Dataset (XLSX)
